# A Survey of Protein Structures from Archaeal Viruses 

**DOI:** 10.3390/life3010118

**Published:** 2013-01-24

**Authors:** Nikki Dellas, C. Martin Lawrence, Mark J. Young

**Affiliations:** 1 Thermal Biology Institute, Montana State University, Bozeman, MT 59717, USA; E-Mails: ndellas@gmail.com (N.D.); lawrence@chemistry.montana.edu (C.M.L.); 2 Department of Plant Sciences and Plant Pathology, Montana State University, Bozeman, MT 59717, USA; 3 Department of Chemistry and Biochemistry, Montana State University, Bozeman, MT 59717, USA; 4 Department of Microbiology, Montana State University, Bozeman, MT 59717, USA

**Keywords:** archaeal virus, thermophile, structural homology, archaea

## Abstract

Viruses that infect the third domain of life, Archaea, are a newly emerging field of interest. To date, all characterized archaeal viruses infect archaea that thrive in extreme conditions, such as halophilic, hyperthermophilic, and methanogenic environments. Viruses in general, especially those replicating in extreme environments, contain highly mosaic genomes with open reading frames (ORFs) whose sequences are often dissimilar to all other known ORFs. It has been estimated that approximately 85% of virally encoded ORFs do not match known sequences in the nucleic acid databases, and this percentage is even higher for archaeal viruses (typically 90%–100%). This statistic suggests that either virus genomes represent a larger segment of sequence space and/or that viruses encode genes of novel fold and/or function. Because the overall three-dimensional fold of a protein evolves more slowly than its sequence, efforts have been geared toward structural characterization of proteins encoded by archaeal viruses in order to gain insight into their potential functions. In this short review, we provide multiple examples where structural characterization of archaeal viral proteins has indeed provided significant functional and evolutionary insight.

## 1. Introduction

Archaeal viruses infect the third domain of life, Archaea. Over 5000 bacterial viruses and eukaryotic viruses have been identified, as compared to the approximately 100 archaeal viruses that have been characterized to date. All identified archaeal viruses infect extremophilic hosts, including acidophiles and hyperthermophiles found in terrestrial hot springs and deep sea vents, alkaliphiles and halophiles that thrive in alkaline and hypersaline environments, and methanogens that survive in anaerobic environments [1,2]. Further investigations will likely uncover archaeal viruses that replicate in mesophilic archaeal hosts [3].

It is well documented that on an evolutionary timescale, the three-dimensional fold of a protein persists longer than its sequence [4]. Thus, in the case of archaeal viral proteins, where there is little sequence similarity at the amino acid level to proteins with known function, efforts have been focused on the utilization of structural homology as a tool to identify distant evolutionary relationships [5]. For example, in many of the cases discussed below, structural characterization has uncovered relationships between archaeal viral proteins and other proteins with known function, some of which belong to different viral families or domains of life. The evolutionary history of archaeal viruses appears very complex, and there is an accumulation of structural and genomic evidence that supports the idea that many genes of a given archaeal viral genome are of different or unknown origin [6,7,8]. 

**Figure 1:**
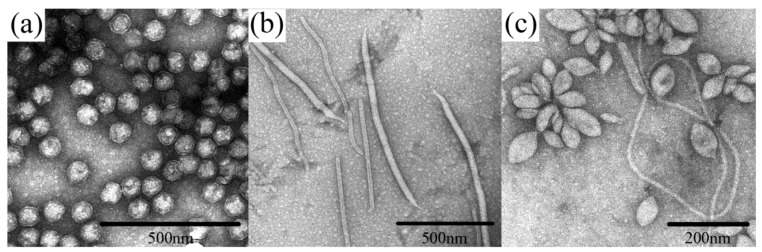
Examples of archaeal virus morphologies. (**a**) *Sulfolobus* turreted icosahedral virus (STIV), belonging to an unclassified Crenarchaeal virus family. (**b**) two viruses are represented: *Sulfolobus islandicus* rod-shaped virus (SIRV) from the family *Rudiviridae*, which are the smaller particles shaped as straight rods, and *Sulfolobus islandicus* filamentous virus (SIFV) belonging to the family *Lipothrixviridae*, which are larger, filamentous particles. (**c**) *Sulfolobus* spindle-shaped virus 1 (SSV1) belonging to the family *Fuselloviridae*. All micrographs are the courtesy of S. Brumfield.

Archaeal viruses that have been identified infect (1) thermoacidophilic or hyperthermophilic members of the phylum Crenarchaeota or (2) halophilic or methanogenic members of the phylum Euryarchaeota. These viruses have been grouped into viral families based on their morphologic and genetic structure [9] (Figure 1). To date, 32 proteins have been structurally characterized from viruses infecting Crenarchaeota belonging to the viral families *Fuselloviridae*, *Lipothrixviridae*, *Rudiviridae*, *Globuloviridae*, *Bicaudaviridae*, and an unclassified viral family to which the viruses *Sulfolobus* turreted icosahedral virus (STIV) and STIV2 belong (Table 1). In comparison, only three cryo-electron microscopy (cryoEM) structures of viruses from Euryarchaeota have been solved, including the icosahedral virus *Haloarcula hispanica* virus (SH1) [10], and two head-tail viruses: (*Haloarcula vallismortis* tailed virus 1 (HVTV-1) and *Halorubrum sodomense* tailed virus 2 (HSTV-2) [11].

**Table 1:** Overview of viruses, viral families, and potential hosts.

Virus	Virus Family	Host
*Haloarcula hispanica* virus (SH1)	unclassified	*Haloarcula hispanica, Halorubrum, Haloferax* [12,13]
*Sulfolobus *turreted icosahedral virus (STIV)	unclassified	*Sulfolobus* [14]
*Acidianus *filamentous virus 1 (AFV1)	*Lipothrixviridae*	*Acidianus* [15]
*Sulfolobus islandicus* rod-shaped virus 1 (SIRV1)	*Rudiviridae*	*Sulfolobus* [16]
*Sulfolobus *spindle-shaped virus 1 (SSV1)	*Fuselloviridae*	*Sulfolobus* [17]

## 2. Discussion

### 2.1. Evolutionary Links Revealed Through Structures of Archaeal Virus Major Capsid Proteins

**Figure 2:**
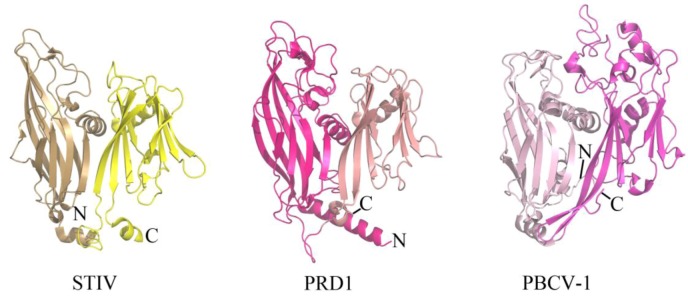
The double β-barrel fold is conserved in a viral lineage found within all three domains of life. The conserved coat protein fold of STIV B345 (PDB ID 2BBD [19]), PRD1 P3 (PDB ID 1GW7 [24]), and PBCV-1 Vp54 (PDB ID 1J5Q [21]) from viruses that infect hosts in Archaea, Bacteria, and Eukarya, respectively. Each jelly roll fold is shaded differently within the three structures.

The coat protein of a virus represents a component of the virion that provides physiochemical stability and protection to the enclosed viral genome. Enveloped viruses contain a host-derived lipid membrane that surrounds the coat protein (as observed in archaeal viruses of the filamentous *Lipothrixviridae*), while other viruses contain an internal lipid membrane. STIV is one such inner lipid membrane-containing virus that belongs to an unclassified viral family within Crenarchaeota. The crystal structure of the major coat protein (MCP) from STIV (B345) reveals a double β-barrel fold, termed the “double jelly roll fold” (Figure 2) [18,19]. Interestingly, the double β-barrel fold is observed in other viruses that infect Bacteria (for example, PRD1 [20]) and Eukarya (for example, PBCV-1 [21]) (Figure 2). In addition to a homologous coat protein fold, these viruses also share other features, such as an internal lipid membrane and conserved genes (for example, a packaging ATPase) [14,22,23]. The cryo-EM structure of SH1, which infects hosts in Euryarchaeota, reveals that its coat protein adopts a single β-barrel fold [10]. It is therefore speculated that SH1 represents an ancient version of the coat protein fold that was then duplicated to generate the double β-barrel fold observed in STIV, PRD1, PBCV-1 and other viruses thought to be part of this lineage [10]. STIV and STIV2 are the only characterized viruses from this lineage that infect thermoacidophilic hosts. There are a variety of features of thermostability observed within the coat protein of STIV, such as its fold compactness and lack of cavities. However, other viruses from this lineage also contain such features and it is not known which of these specifically contribute to STIV's ability to maintain structural integrity within a thermophilic environment [19].

**Figure 3:**
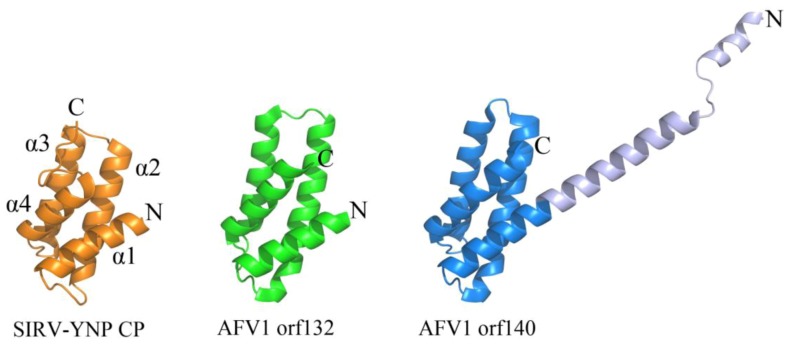
A conserved 4-helix bundle arrangement found in archaeal viruses from *Lipothrixviridae* and *Rudiviridae*. The two structural proteins from AFV1, orf132 (PDB ID 3FBL [25]) and orf140 (PDB ID 3FBZ [25]) adopt the same 4 α-helix bundle arrangements as the C-terminal domain of the single coat protein from SIRV-YNP (PDB ID 3F2E [30]). It is noteworthy that the first 50 amino acids from AFV1 orf132 are absent from the x-ray data. The N-terminal and C-terminal ends of each protein structure are labeled with “N” and “C”, respectively. The α-helices are labeled for SIRV-YNP CP and follow the same arrangement in the other two protein structures.

Certain viruses belonging to *Lipothrixviridae* and *Rudiviridae* have also demonstrated a conserved coat protein fold. In fact, a viral order (*Ligamenvirales*) encompassing both of these families has been proposed on the basis of genomic similarities between members of these two families that extend beyond conservation of the gene encoding the coat protein [9]. Viral members of both families are linear in morphology; however, those from *Lipothrixviridae* are enveloped and exist as (400–1,950) × 24–38 nm flexible, filamentous particles, while those from *Rudiviridae* are (610–900) × 23 nm non-enveloped, stiff, and rod-shaped in morphology [9]. *Acidianus* filamentous virus 1 (AFV1) belongs to *Lipothrixviridae* and encodes two structural proteins (orf132 and orf140), both of which contain an anti-parallel four-helix bundle fold that is structurally homologous to the C-terminal domain of the single MCP from *Sulfolobus islandicus* rod-shaped virus from Yellowstone National Park (SIRV-YNP) (Figure 3) [25]. Interestingly, tobacco mosaic virus, a thermostable, eukaryotic positive strand RNA virus, utilizes a heavily decorated 4-helix bundle to assemble a rod-like helical structure [26]. In contrast to tobacco mosaic virus, it is not yet known how these archaeal proteins are assembled into their presumably helical rod- or filamentous forms. However, a model has been proposed for coat protein assembly within AFV1: double-stranded DNA is thought to wrap around the positively charged protein AFV1 orf132, while AFV1 orf140 is proposed to interact with exterior of the DNA-protein bundle through its N-terminus and with the lipid membrane through its hydrophobic C-terminal helix [25].

### 2.2. Common DNA-Binding Motifs Observed in Proteins from Archaeal Viruses

**Figure 4:**
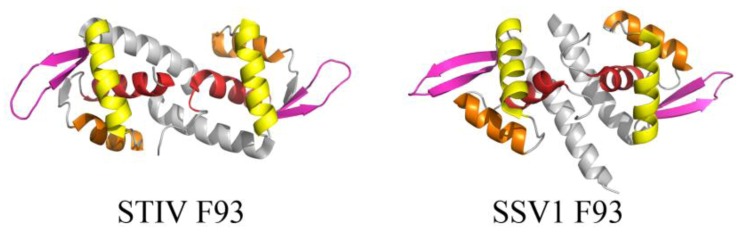
wHTH motif found in two structural homologs from STIV and SSV1. The overall folds of STIV F93 (left, PDB ID 2CO5 [28]) and SSV1 F93 (right, PDB ID 1TBX [29]) highlight the components of the wHTH motif, including (in order of N- to C-terminal) α1 (red), α2 (orange), α3 (the recognition helix, yellow), and a β-sheet (pink, comprising β2, β3 for STIV F93 and β1, β2 for SSV1 F93).

Many of the published structures of archaeal viral proteins reveal DNA-binding motifs that are found in other organisms, such as the winged helix-turn-helix (wHTH) and the ribbon helix-helix (RHH) folds. The core of the wHTH fold is composed of a right handed three helix bundle followed by two β-strands, forming a 2 stranded antiparallel β-sheet known as the wing (Figure 4). The third helix of the three-helix bundle is the recognition helix, which inserts into the major groove of DNA [27], while the wing is often involved in nonspecific interactions with the ribose phosphate backbone. The wHTH motif is present in all three domains of life and often functions as a DNA recognition component of various transcription factors. The fold has also been found less frequently in proteins within RNA metabolism and those that are involved in protein-protein interactions [27]. Many archaeal virus proteins that adopt the wHTH motif are thus suggested to play roles in transcriptional regulation, although for many of these, specific binding sites within the viral or host genomes have yet to be characterized. Examples include F93 from STIV (unclassified viral family) and F93 from *Sulfolobus* spindle-shaped virus 1 (SSV1) (*Fuselloviridae*) (Figure 4). These dimeric wHTH proteins are structurally homologous to the prokaryotic MarR/SlyA protein family of transcription regulators [28,29] and are therefore expected to recognize (pseudo-) palindromic DNA targets.

**Figure 5:**
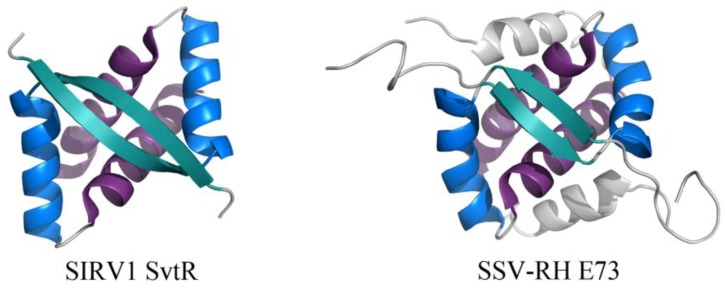
RHH motifs in two unrelated proteins encoded by viruses from *Fuselloviridae* and *Rudiviridae*. The overall folds of SIRV1 SvtR (left, PDB ID 2KEL [33]) and SSV-RH E73 (right, PDB ID 4AAI [32]) are shown. The components of the RHH fold are highlighted for each dimer, including (in order of N- to C-terminal) β1 (turquoise), α1 (blue), and α2 (purple). SSV-RH E73 has an elaboration of the RHH fold, containing an additional α-helix (α3, colored gray) that may enhance the stability of its dimer and/or contribute to an additional ligand binding site.

The RHH fold is well named, beginning with a β-strand that precedes two α-helices. This β-strand interacts with the β-strand of another subunit, forming an anti-parallel two-stranded β-sheet that inserts into the major groove of DNA (Figure 5) [31]. While the wHTH motif is found in all domains of life, the RHH motif is found strictly in prokaryotes. Archaeal viral proteins that adopt the RHH fold (or elaborated versions of this) include E73 from *Sulfolobus* spindle-shaped virus from Ragged Hills (SSV-RH, *Fuselloviridae*) [32] and SvtR from SIRV1 (*Rudiviridae*) (Figure 5) [33]. SvtR is currently the best-characterized example of an archaeal virus protein containing the RHH motif. It is structurally homologous to bacterial RHH proteins and was determined to bind four target sequences from the SIRV1 genome. Target sequences include those preceding the coding region for its own gene as well as that for the coat protein; transcription of both the coat protein gene and its own gene were blocked by SvtR in an *in vitro* transcription assay [33].

### 2.3. Structurally Characterized Enzymes of Archaeal Viruses

**Figure 6:**
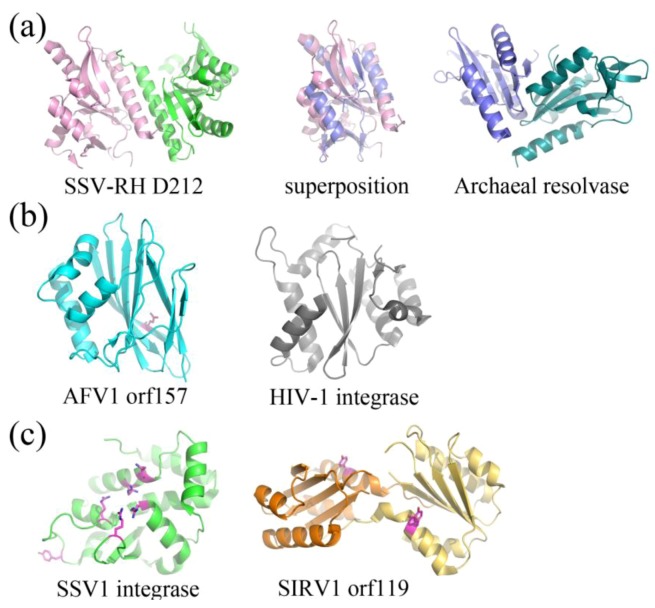
Fold Topologies from archaeal viral enzymes involved in nucleic acid metabolism. **(a)** SSV-RH D212 (left, PDB ID 2W8M [35]) with a fold that is similar to those of members belonging to the PD-D/EXK nuclease superfamily. An archaeal member of this family, HJC Holliday junction resolving enzyme from *Sulfolobus solfataricus* (right, PDB ID 1HH1 [40]) aligns quite well with a single D212 subunit (middle), however the dimer interfaces are substantially different for the two. Each subunit within the dimer is colored differently. **(b)** AFV1 orf157 (left, PDB ID 3II2 [34]) very distantly resembles the two-layer sandwich fold of the phosphonucleotidyl superfamily to which HIV1-integrase belongs (right, PDB ID 1ITG [41]), however it only has one layer of α-helices and not two. A catalytic residue, Glu86, is colored magenta. **(c) **SSV1 integrase (left, PDB ID 3VCF [36]) shares a conserved fold and catalytic residues with members belonging to the tyrosine recombinase family (highlighted in magenta), including the catalytic pentad (cluster of amino acids in the center of the protein) and the tyrosine nucleophile (distal to the active site). SIRV1 orf119 (right, PDB ID 2X3G [38]) has been identified as a novel type of Rep protein with the conserved fold of the superfamily II rep protein group. It shares conserved features with members of this group, including a conserved catalytic tyrosine (highlighted in magenta). Each subunit for the Rep protein dimer is colored differently.

**Figure 7:**
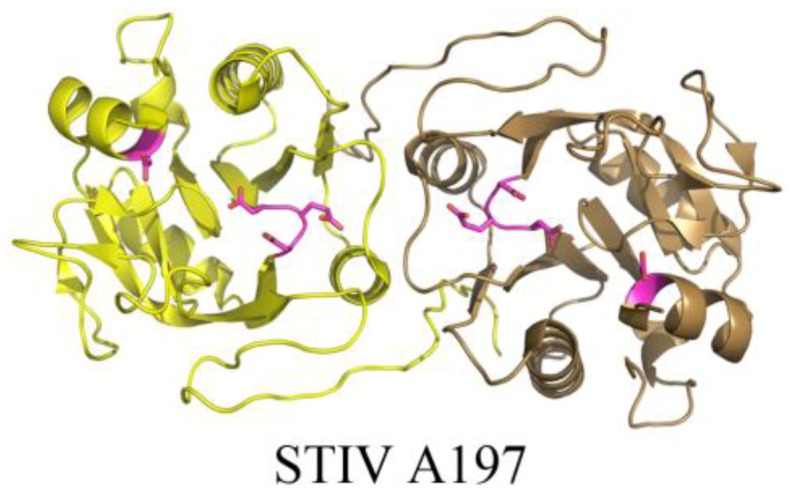
The crystal structure of a putative glycosyltransferase from the archaeal virus, STIV. STIV A197 (PDB ID 2C0N [39]) adopts the glycosyltransferase GT-A family fold and contains many of the conserved catalytic residues seen throughout the family, including the DXD motif (located in a loop, colored magenta), a catalytic base, (Asp151, located in a helix, colored magenta), and a conserved manganese binding site (not depicted).

To date, there have been five structurally characterized proteins that are thought to serve an enzymatic role in the context of a viral infection. Four out of five structurally characterized enzymes from archaeal viruses are involved in nucleic acid metabolism. These include crystal structures of two unrelated nucleases [34,35] as well as the SSV1 viral integrase (SSV1Int) [36,37] and a Rep protein (Figure 6) [38]. In addition, structural studies have also identified a putative gylcosyltransferase (Figure 7) [39]. Each of these enzymes is suggested to have a different evolutionary origin, highlighting the putative complexity and mosaicity of archaeal viral genomes.

Putative and characterized nucleases include SSV-RH D212 (*Fuselloviridae*) and AFV1 orf157 (*Lipothrixviridae*), respectively. SSV-RH D212 adopts the PD-D/EXK nuclease superfamily fold, however its activity remains uncharacterized and is therefore only a putative nuclease. This protein most closely resembles an archaeal Holliday junction resolvase. The full-length version is not active in the traditional Holliday junction cleavage assay and a clipped version only shows very low levels of metal-dependent nuclease activity, suggesting that the DNA binding surfaces are distinct between SSV-RH D212 and its closest homologs (Figure 6a) [35]. AFV1 orf157 very distantly resembles a trimmed down version of the two-layer sandwich fold of HIV1-integrase from the phosphonucleotidyl superfamily, which successfully guided experiments toward the functional characterization of orf157 as a nuclease (Figure 6b) [34].

The structurally and functionally characterized viral integrase from SSV1 (SSV1Int, *Fuselloviridae*) is a member of the tyrosine recombinase superfamily (Figure 6c). Interestingly, the biochemical [42] and structural analyses suggests that during strand exchange and cleavage, the enzyme assembles its active site in *trans*, which is consistent with mechanisms utilized by eukaryotic and not bacterial tyrosine recombinases [36,37]. SIRV1 orf119 (*Rudiviridae*) adopts a fold that is similar to superfamily II of Rep proteins, involved in the initiation of genomic replication (Figure 6c) [38]. Orf119 implements a novel “flip-flop” mechanism that is unique to conventional rolling circle replication (RCR) or rolling hairpin replication (RHR). This flip-flop mechanism may be employed by Rep proteins in other viruses that have closed, linear dsDNA genomes, such as members of the eukaryotic viral family, *Poxviridae* and bacteriophage N15 [38].

The crystal structure of STIV A197 reveals a putative glycosyltransferase adopting the GT-A fold whose closest structural homologs mainly consist of eukaryotic glycosyltransferases (Figure 7) [39]. Because the major coat protein of STIV is known to be glycosylated [14] and the virus assembles in the cytosol [43], A197 is a strong candidate for this activity, however it presently remains uncharacterized.

## 3. Conclusions

Structural characterization of proteins from archaeal viruses has lead to several conclusions. One conclusion is that certain archaeal viruses may be extant versions of ancient viral lineages. For example, the single β-barrel fold from SH1 [10] and double β-barrel fold from STIV [19] share homologs in viruses infecting Bacteria and Eukarya, suggesting that this fold was present in a common ancestor that existed before the domains of life emerged [19,20]. While viruses from this lineage share certain similarities, the majority of the genes are not obviously conserved by sequence homology. This observation supports the idea that archaeal viruses, like most viruses in general, act as mobile genetic elements and can potentially gain, lose, and transfer genetic material quite easily [44]. Collectively, the examples of structurally characterized archaeal viral enzymes also emphasize the fact that the genes comprising an archaeal virus genome are mosaic and may originate from different domains of life [8]. For example, the tyrosine recombinase from SSV1 employs a mechanism that parallels that utilized for the same enzyme in eukaryotes [36,37]). The fact that archaeal viruses (as well as viruses from Bacteria and Eukarya) code for and maintain a high abundance of DNA-binding motifs suggests that it is an indispensable feature that is present among all domains of life [8,27]. 

The majority of structurally characterized archaeal virus proteins infect hyperthermophilic archaea of the phylum Crenarchaeota. It is therefore not surprising that many structural studies of proteins from archaeal viruses report features of thermostability including compactness of fold [45], absence of cavities [46], a high number of salt bridges [46], a high ratio of charged to uncharged residues [45,47], short loops [45], oligomerization with (in some cases) extensive subunit interfaces [32,33] and the presence of disulfide bonds [28,36,37,46,48,49,50]. In contrast to the cellular proteins of mesophilic organisms, which do not generally utilize disulfide bonds, there is strong genomic [28,48,51,52,53] and metagenomic [5] evidence for the use of stabilizing disulfides in hyperthermophiles and their viruses.

Currently, there exist a relatively small number of archaeal viral protein structures compared to those in the bacterial and eukaryotic viral domains. Archaeal viruses therefore remain the largest group of unexplored territory in the realm of protein structural and functional characterization. Given the vast sequence diversity of archaeal viral proteins and lack of identifiable protein homologs, it is tempting to speculate that fold novelty would be commonly observed for structures of these proteins. While there are several structures of archaeal viral proteins with novel folds [47,50,54,55], the majority that have been characterized thus far share structural homology to proteins of known function. However, it is likely that the small subset of archaeal viral protein structures biases our current perspective, and that as more of these proteins are structurally characterized, fold novelty may become more commonly observed from viruses infecting this domain of life. Therefore, in addition to the examples discussed herein of fold conservation masked by sequence diversity, future examples of fold novelty may also explain the higher than usual level of unidentifiable genes in archaeal virus genomes. 

Structural studies of archaeal viral proteins have facilitated the investigation of distant evolutionary relationships and the identification of functionally characterized structural homologs. High levels of sequence divergence have hindered the ability to rely on sequence for functional annotation, and as a consequence, structural characterization has become invaluable as a tool for archaeal viral protein characterization. The vast amount of sequence space covered by archaeal viruses may be a function of evolutionary distance and selective pressures (such as high temperature) imposed on the virus to maintain viability in the context of both the hyperthermophilic environment and the host. As proven through structural annotation, such sequences often mask a conserved protein fold, however examples of fold novelty also exist and most likely contribute to the high levels of sequence diversity observed among archaeal viral genes. 
